# Superior Vena Cava Syndrome Revealing Sarcoidosis a Decade Later: A Case Report

**DOI:** 10.7759/cureus.69238

**Published:** 2024-09-11

**Authors:** Aziza Rhanim, Leila Achachi, Hajar Benataya, Mustapha El Ftouh, Laila Herrak

**Affiliations:** 1 Department of Pulmonology, Ibn Sina University Hospital Center, Faculty of Medicine and Pharmacy, Mohamed V University, Rabat, MAR

**Keywords:** granuloma, lymphadenopathy, sarcoidosis, superior vena cava syndrome, vessel

## Abstract

Sarcoidosis is a systemic disease of unknown origin, characterized by the formation of immune granulomas in various organs. The occurrence of superior vena cava syndrome (SVCS) remains very rare. We report the case of a 54-year-old patient treated for cervical lymph node tuberculosis in 2010. Three years later, she presented with SVCS of undetermined etiology. In 2023, mediastino-pulmonary sarcoidosis of type II was confirmed in her. The patient remained clinically stable under surveillance and did not receive any specific treatment for sarcoidosis. Superior vena cava syndrome is often associated with malignancy. Benign etiologies, particularly sarcoidosis, although rare, should be investigated

## Introduction

Sarcoidosis is a systemic disease of unknown origin, characterized by the formation of immune granulomas in various organs, with a preference for the lungs and the mediastinal lymphatic system. Specific vascular manifestations in sarcoidosis include necrotizing sarcoid angiitis, pulmonary embolus in the setting of antiphospholipid antibody syndrome, extrinsic compression of the main pulmonary arteries, and pulmonary hypertension [1]. The occurrence of superior vena cava syndrome (SVCS) remains extremely rare, only nine cases have been reported in the literature. We report a case of superior vena cava syndrome with an undetermined etiology despite extensive investigation, in a patient diagnosed with mediastino-pulmonary sarcoidosis 10 years later.

## Case presentation

The patient is a 54-year-old woman who was treated for isolated cervical lymph node tuberculosis in 2010. Three years later, she developed superior vena cava syndrome. A chest CT scan revealed circumferential tissue thickening of the superior vena cava, measuring 11 mm in thickness and located 3 cm above its junction with the right atrium, extending 3.5 cm in height, with no parenchymal lesions or mediastinal adenopathy. An extensive etiological testing did not identify a specific cause, and the patient was placed on anticoagulant therapy (low molecular weight heparin relayed by warfarin) with symptom improvement (facial swelling and dyspnea). Lost of sight until 2017, a new injected chest scanner confirmed the same radiological aspect. A second etiological investigation did not reveal an obvious cause, and the patient continued anticoagulant therapy with warfarin, with regular follow-up and monitoring.

In September 2023, the patient presented with inflammatory arthralgia of the large joints without any other associated symptoms. On clinical examination, the patient was in good general condition, with a performance score of 0, normal respiration at 18 cycles per minute, a heart rate of 75 beats per minute, and an oxygen saturation of 96%. We noticed an anterior thoracic collateral venous circulation, a slight filling of the supraclavicular fossae, and jugular vein engorgement. The pleuropulmonary and cardiovascular examinations were normal, as were the peripheral lymph nodes.

**Figure 1:**
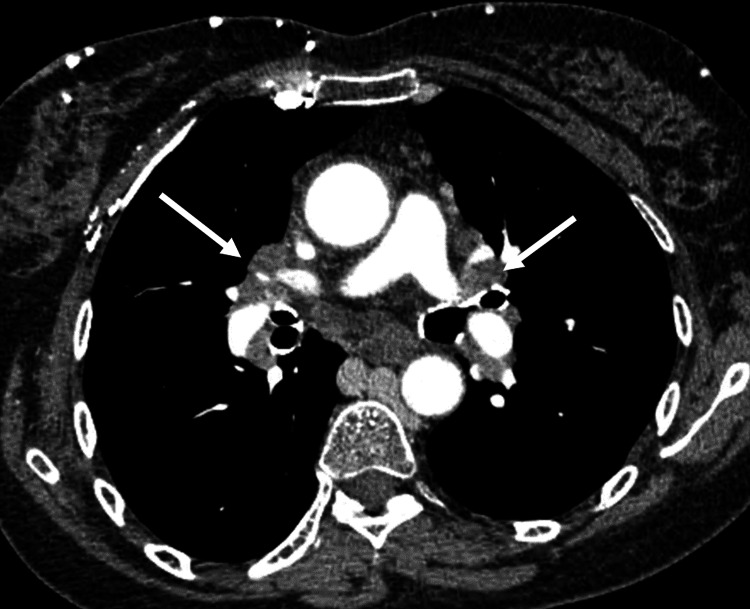
Chest CT scan, mediastinal window, axial section after iodinated contrast injection with acquisition at 35 seconds: Bilateral symmetric non-compressive hilar mediastinal lymphadenopathy

**Figure 2:**
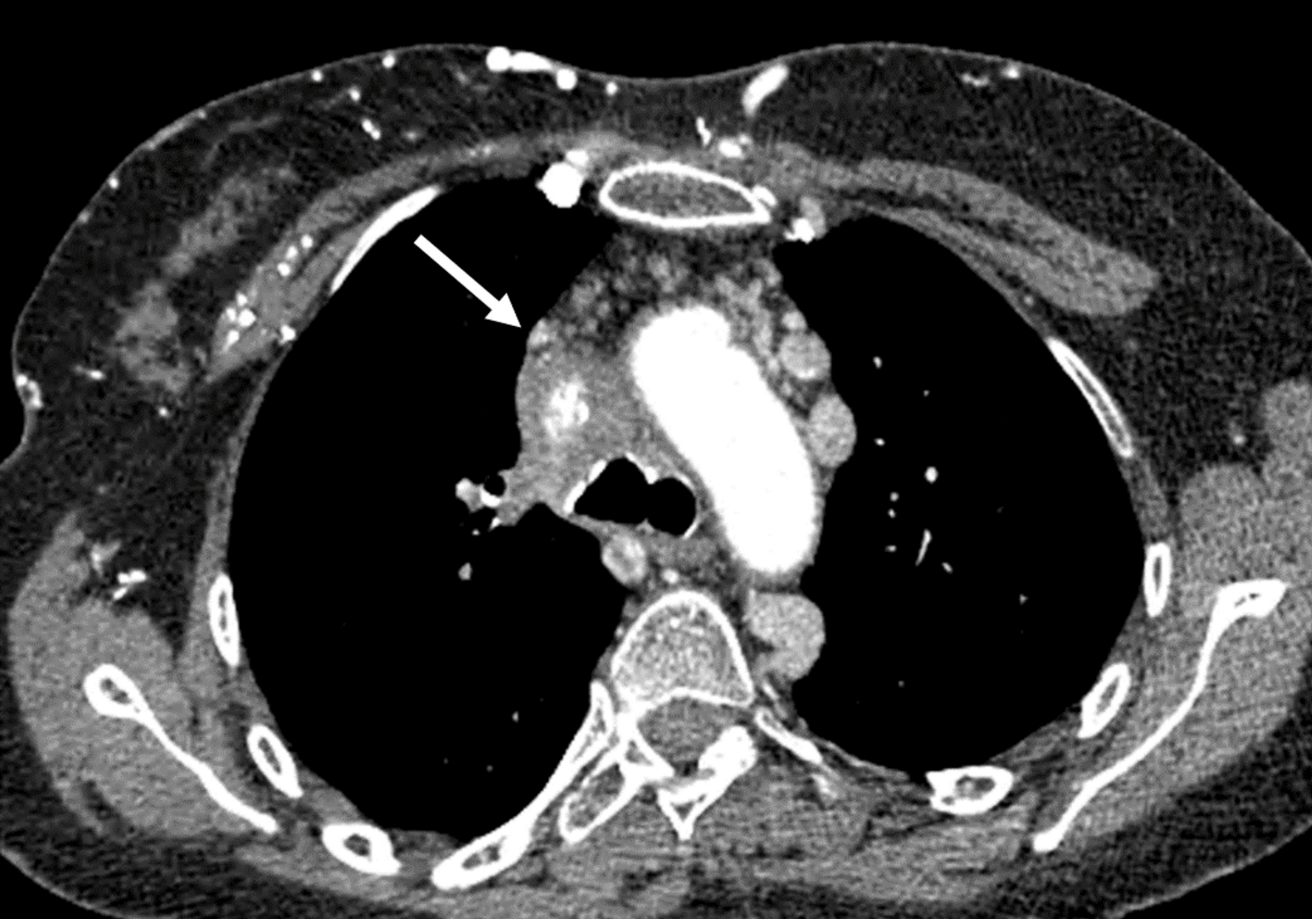
Chest CT scan, mediastinal window, axial section after iodinated contrast injection with acquisition at 35 seconds: Circumferential parietal thickening of the SVC + CVC thoracic wall SVC: superior vena cava; CVC: collateral venous circulation

An injected chest CT was performed, which showed bilateral symmetrical mediastinal-hilar lymphadenopathy, the largest measuring 21 mm, located subcarinally without pulmonary parenchymal involvement (Figure 1), with the complete thrombosis of the superior vena cava up to its termination, along with calcifications and a partial thrombosis of the internal jugular veins extending to both brachiocephalic trunks (Figure 2).

Laboratory tests (complete blood count, renal function tests, liver function tests, calcium levels, 24-hour proteinuria, and lactate dehydrogenase (LDH) levels) were normal. Protein electrophoresis showed an inflammatory syndrome with high levels of alpha-1 and alpha-2 proteins. The Xpert genetic test on sputum was negative, as were the immunological exams including anti-nuclear antibodies, anti-DNA antibodies, anti-anti-cyclic citrullinated peptide (CCP) antibodies, and rheumatoid factor. Bronchoscopy revealed mild bilateral inflammatory changes of the first degree with slightly thickened spur formations. The histopathological study of the staged bronchial biopsies and the biopsy of accessory salivary glands showed an epithelioid giant cell granuloma without caseous necrosis, and the BK culture on bronchial biopsy was negative. Pulmonary function tests were normal. The diagnosis of sarcoidosis was retained.

**Figure 3:**
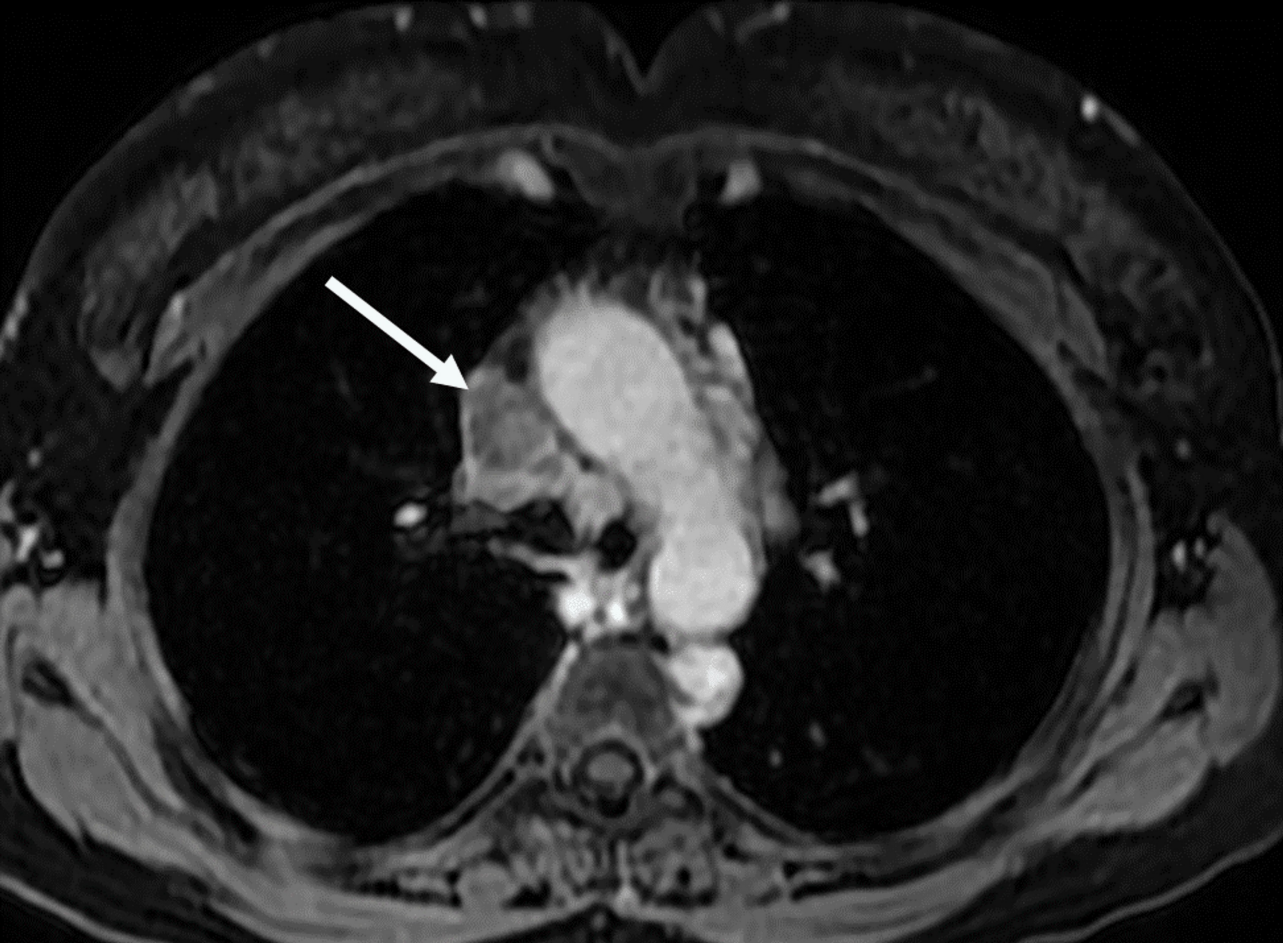
Thoracic MRI in T1 fat-saturated sequence with gadolinium injection: Circumferential parietal thickening of the SVC that moderately enhances with gadolinium injection, suggesting an inflammatory process SVC: superior vena cava

**Figure 4:**
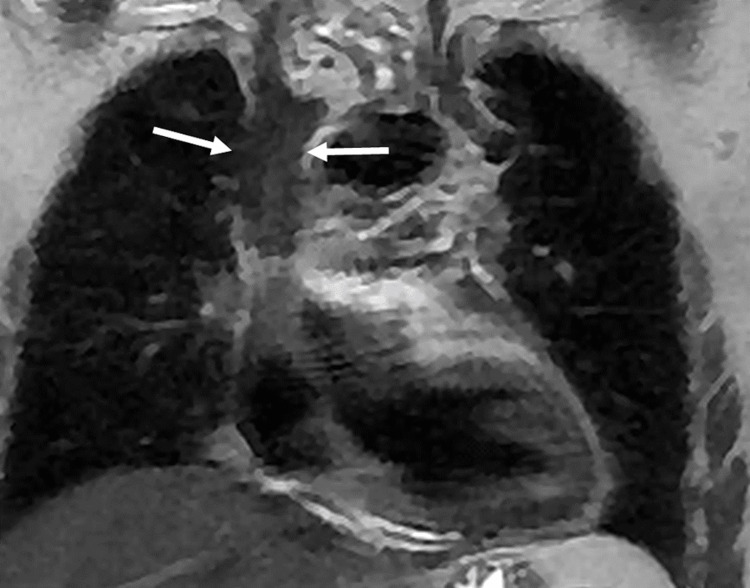
Thoracic MRI in coronal T2 sequence without fat saturation: Extensive parietal thickening of the SVC SVC: superior vena cava

To establish a link between superior vena cava syndrome and sarcoidosis, an additional test including a thoracic MR angiography was performed, showing inflammatory wall thickening of the superior vena cava responsible for the venous thrombosis (Figures 3, 4). A PET scan was also carried out to assess disease activity in the superior vena cava and to search for other affected sites, it revealed a wall thickening of the superior vena cava with some calcifications, without pathological hypermetabolism, and active lymphadenopathy above and below the diaphragm with some bilateral pulmonary micronodules.

The diagnosis of asymptomatic stage II mediastino-pulmonary sarcoidosis with superior vena cava involvement was confirmed, and a specific therapeutic abstention for the disease, maintaining thrombosis treatment.

Follow-up at three and six months showed no clinical, functional, or radiological deterioration.

## Discussion

Sarcoidosis is a common granulomatous disease affecting all organs, with preferential locations such as lungs, skin, eyes, superficial lymph nodes, and liver. However, some atypical manifestations, though very rare, can pose differential diagnostic issues. The aspect of superior vena cava syndrome in the context of sarcoidosis is extremely rare, with the most common causes being bronchopulmonary cancer and lymphoma [2].

In sarcoidosis, superior vena cava syndrome is often related to compression by hypertrophied lymph nodes [3]. Vein thrombosis is also a rare manifestation; the exact mechanism remains unknown [4]. Only nine cases of superior vena cava syndrome associated with sarcoidosis have been reported in the literature [5-13], and in some cases, the superior vena cava syndrome was the first clinical manifestation of sarcoidosis. In our particular clinical case, the superior vena cava syndrome occurred ten years before the diagnosis of sarcoidosis, despite repeated extensive initial investigations. The hypothesis of specific vascular involvement by sarcoidosis was suggested due to the absence of compressive lymph nodes and imaging results showing circumferential thickening of the superior vena cava, later confirmed by MR angiography and PET scan.

Granulomatous involvement of small and medium-sized vessels is common in sarcoidosis, often asymptomatic and without significant functional impact [3]. In contrast, the involvement of large vessels by sarcoid granulomas and the resulting obstruction is very rare, as in our patient's case. For instance, in a case published by Narayan in 1998 [11], the obstruction of the superior vena cava was present without inducing a mass syndrome, with typical non-caseating granulomas of sarcoidosis at histopathological examination. Other cases of granulomatous angiitis associated with sarcoidosis have been described [14].

Therapeutic management varies depending on cases, with the majority showing regression of superior vena cava syndrome after corticosteroid treatment [5, 7, 8, 10, 11]. Two cases regressed without medical or surgical treatment [9, 13], while only one case required surgical intervention along with corticosteroids [11].

## Conclusions

This case highlights the diagnostic complexity of sarcoidosis with atypical manifestations such as superior vena cava syndrome. Despite extensive investigations, the etiology of SVCS remained undetermined for a decade until mediastino-pulmonary sarcoidosis was diagnosed. The superior vena cava thrombosis due to inflammatory thickening, confirmed by MR angiography and PET scan, underscores a rare specific vascular involvement. This case emphasizes the importance of considering sarcoidosis in benign SVCS diagnoses and the effectiveness of a conservative management approach focused on treating the thrombosis.
